# Obstetric Hysterectomy at a Greek Tertiary Referral Center: Differences in Maternal and Neonatal Outcomes Between Elective and Urgent Procedures

**DOI:** 10.3390/jcm15197738

**Published:** 2026-10-08

**Authors:** Stefanos Flindris, Margioula-Siarkou Chrysoula, Konstantinos Christopoulos, Georgia Margioula-Siarkou, Ioannis Chatzikalogiannis, Michail Kalinderis, Alexandros Traianos, Elif Empliouk, Konstantinos Pantazis, Alexandros Sotiriadis, Konstantinos Dinas, Stamatios Petousis

**Affiliations:** 1 2nd Department of Obstetrics and Gynecology, Aristotle University of Thessaloniki, Faculty of Health Sciences, Medical School, General Hospital of Thessaloniki “Ippokratio”, 54642 Thessaloniki, Greece; 2 Department of Hygiene and Epidemiology, University of Ioannina, Medical School, 45500 Ioannina, Greece; 3 1st Department of Obstetrics and Gynecology, Aristotle University of Thessaloniki, Faculty of Health Sciences, Medical School, General Hospital of Thessaloniki “Papageorgiou”, 56403 Thessaloniki, Greece

**Keywords:** placenta accreta spectrum, obstetric hysterectomy, elective surgery, emergency hysterectomy, postpartum hemorrhage, maternal morbidity, neonatal outcomes

## Abstract

**Background:** Obstetric hysterectomy remains a life-saving intervention for placenta accreta spectrum and uncontrolled obstetric hemorrhage. Evidence regarding the outcomes of planned versus urgent obstetric hysterectomy in contemporary tertiary practice in Greece remains limited. We compared maternal and neonatal outcomes between elective and urgent procedures at a tertiary referral center in Thessaloniki. **Methods:** We retrospectively reviewed 30 women who underwent obstetric hysterectomy between January 2020 and June 2025. The elective group included women with prenatal PAS diagnosis and planned delivery at ≥34 weeks (n = 17), whereas the urgent group included women requiring unplanned delivery for hemorrhage or active labor without prior surgical preparation (n = 13). Maternal characteristics, intraoperative findings, transfusion requirements, complications, hospital stay, and neonatal outcomes were compared using bivariate statistical tests. **Results:** Maternal age was similar between groups (36.1 ± 6.1 vs. 37.9 ± 6.8 years; *p* = 0.44). Elective cases had higher parity (median 3 vs. 1; *p* = 0.002), had more previous cesarean deliveries (median 2 vs. 0; *p* = 0.002), and were more frequently diagnosed with placenta previa (*p* = 0.027). Uterine atony occurred more often in urgent cases (*p* < 0.001). Bakri balloon tamponade was used only in urgent cases and failed to control hemorrhage (*p* < 0.001). Operative time, transfusion requirement, hospital stay, and urinary tract injury rates were comparable. No maternal deaths occurred. Birth weight, Apgar scores, and NICU admission rates were also similar. **Conclusions:** Planned obstetric hysterectomy within a multidisciplinary setting was associated with fewer emergency hemorrhage-control interventions, while major perioperative and neonatal outcomes were comparable between groups.

## 1. Introduction

Obstetric hysterectomy (OH) is the surgical removal of the uterus and cervix, with or without unilateral or bilateral adnexectomy, which is performed after vaginal delivery or during cesarean section in the puerperium. Its main indication is life-threatening obstetric hemorrhage refractory to conservative management, as well as abnormal placentation (placenta accreta or previa), placental abruption, and uterine atony [1,2]. The FIGO consensus on nonconservative surgical management reports maternal mortality rates ranging from approximately 1% to 7% for the placenta accreta spectrum [3]. Although OH permanently eliminates fertility, emergency obstetric hysterectomy (EOH) remains the definitive intervention for severe postpartum hemorrhage and a vital safeguard against maternal mortality [2].

The global incidence of EOH is estimated at 1.1 per 1000 live births, with notable disparities between regions. Approximately 3 per 1000 in lower-middle-income countries versus 0.7 per 1000 in high-income settings [2,4]. Despite its infrequency, EOH demands prompt decision-making, advanced surgical expertise, and seamless coordination among obstetricians, anesthesiologists, blood-bank services, and critical-care teams. Insufficient preparation, whether from limited surgical experience, delays in blood product availability, or suboptimal anesthesia, amplifies morbidity and mortality risks [5].

In a tertiary center in Greece, placenta accreta accounted for 51.1% of EOH cases, and placenta previa for 26.7% [6]. These finding reflect a worldwide trend of rising OH rates parallel to the increasing rate of cesarean deliveries and a consequent surge in placenta accreta spectrum disorders [6]. Patients undergoing OH remain at substantial risk for severe complications, including disseminated intravascular coagulation, acute renal failure, ischemic cardiac injury, encephalopathy, septicemia, and death [5].

Crucially, 33% to 67% of placenta accreta spectrum cases go undiagnosed until delivery, precluding pre-planning in specialized multidisciplinary centers and thereby elevating maternal risk [7,8,9]. Elective obstetric hysterectomy in high-risk patients enables essential preoperative optimization including ureteric stent placement, blood bank mobilization, and the assembly of specialized interdisciplinary teams. Consequently, this planned approach is consistently associated with reduced intraoperative blood loss, fewer blood transfusions, and lower postoperative complication rates compared to emergency interventions [5,10,11]. Given that hemorrhage from morbidly adherent placenta can be rapid and catastrophic, a preemptive, protocol-driven strategy, and timely decision to proceed with OH are paramount to improving maternal outcomes [12].

The aim of this study is to compare baseline maternal characteristics, placental pathology, perioperative management, and maternal and neonatal outcomes between patients that underwent elective or urgent OH at a Greek tertiary referral center.

## 2. Materials and Methods

### 2.1. Study Design and Patient Sample

We conducted a retrospective study of women who underwent obstetric hysterectomy at the Second Department of Obstetrics and Gynecology, Medical School, Aristotle University of Thessaloniki, General Hospital of Thessaloniki “Ippokratio” Greece, between January 2020 and June 2025. The study was conducted in accordance with the Declaration of Helsinki. Informed consent was waived, given the retrospective chart review design of the study. The institutional review board approved the study protocol (24243/2-6-2025-1) and waived the requirement for informed consent due to the retrospective design. Eligible patients were identified through the obstetric and pathology databases for placenta accreta, increta, and percreta, as well as uterine atony. Importantly, both planned and urgent hysterectomies were undertaken by the specialized Gynecologic Oncology surgical team of the Department, reducing potential confounding related to differences in operator expertise.

### 2.2. Data Collection

Clinical data were abstracted from electronic medical records, including maternal demographics (age, parity, prior cesarean deliveries), obstetric history (placenta previa, hypertensive disorders, gestational diabetes, fetal growth restriction), and use of assisted reproductive technology. PAS severity was classified intraoperatively and confirmed on histopathology according to the FIGO grading system (accreta, increta, percreta). Cases were stratified into an “elective” group (planned delivery at ≥34 weeks’ gestation without labor or bleeding) and an “urgent” group (unplanned delivery due to antepartum hemorrhage, labor, or hemodynamic instability). We recorded perioperative variables (operating time, estimated blood loss, pre- and postoperative hemoglobin), surgical interventions (hysterectomy, salpingo-oophorectomy, uterine artery ligation, Bakri balloon placement), transfusion requirements, and maternal complications (organ injury, intensive care admission, reoperation, coagulopathy).

### 2.3. Definition of Radical Hysterectomy Case

The term “radical hysterectomy” applies to one urgent case in our sample of FIGO grade 3c placenta percreta with parametrial and uterine arterial invasion. As previously reported in detail, the operation was classified as a Type C radical abdominal hysterectomy according to the Querleu–Morrow system and included resection of the uterus/cervix with involved parametrial tissue, bilateral salpingo-oophorectomy, and a segment of the upper vagina. The indication was uncontrolled PAS-related hemorrhage, not uterine atony or malignancy. It was not a 1:1 application of the Soleymani–Alazzam–Collins technique because bilateral internal iliac artery ligation was not performed as an initial devascularization step [13,14,15].

### 2.4. Prenatal Diagnosis of Placenta Accreta Spectrum

Prenatal suspicion of PAS was based primarily on targeted trans-abdominal and/or transvaginal ultrasonography incorporating gray-scale and Color Doppler assessment, in accordance with established international diagnostic descriptors. Sonographic features considered suggestive of PAS included abnormal placental lacunae, loss or irregularity of the retroplacental hypoechoic “clear zone,” marked myometrial thinning, disruption of the uterovesical interface, placental bulging, and increased subplacental or uterovesical vascularity, including turbulent lacunar flow and bridging vessels [16]. Ultrasound examinations were performed and interpreted by gynecologists with substantial expertise in prenatal imaging and PAS assessment. Color Doppler was routinely incorporated into the targeted evaluation, whereas magnetic resonance imaging was used selectively in cases with equivocal ultra-sonographic findings or when deeper invasion, particularly placenta percreta or posterior placental involvement, required further anatomical characterization; MRI examinations were interpreted by experienced radiologists [16,17,18]. This approach is consistent with ACOG/SMFM recommendations, which regard expert ultrasonography as the primary diagnostic modality and MRI as an adjunct rather than a first-line investigation [17,19]. Prenatal imaging findings were subsequently correlated with intraoperative findings and definitive histopathological examination to assess imaging–surgical–pathological concordance. The intraoperative extent of placental invasion was classified according to the FIGO clinical grading system for PAS, with histopathologic confirmation whenever hysterectomy specimens were available [3,20].

### 2.5. Perioperative Hemorrhage and Hemostatic Management

Perioperative management of major obstetric hemorrhage followed a standardized, multidisciplinary institutional protocol. All thirty patients received intravenous tranexamic acid (1 g in 100 mL of 0.9% normal saline, repeated after 30 min if necessary). Following conventional surgical hemostasis, oxidized regenerated cellulose powder and a human fibrinogen/thrombin-coated collagen patch were applied as adjunctive topical hemostats in all cases. When clinically indicated, massive transfusion protocols and additional hemostatic therapies were initiated by the anesthesiology team based on hemodynamic status, ongoing blood loss, coagulation profiles, and current international guidelines. However, detailed patient-level data regarding massive transfusion activation and the specific use of additional hemostatic products were not consistently available for quantitative analysis [21].

### 2.6. Neonatal Outcomes

For neonates, we collected birth weight, sex, Apgar scores at 1 and 5 min, and neonatal intensive care unit (NICU) admission. Fetal growth restriction was defined as birth weight below the 10th percentile for gestational age.

### 2.7. Statistical Analysis

Differences between patients with elective and urgent hysterectomy were examined using bivariate statistical tests. These concerned baseline maternal characteristics, placental pathology, perioperative management, and maternal and neonatal outcomes. For continuous variables, descriptive statistics are given as the mean and standard deviation, and sample comparisons were performed using Welch two-sample *t*-tests when normal or Wilcoxon’s rank sum tests with continuity correction when non-normal. For count variables, descriptive statistics are given as the median and interquartile range (IQR), and comparisons were performed with Wilcoxon’s rank sum tests with continuity correction. For binary outcomes, descriptive statistics are given as counts and percentages, and comparisons were performed with Pearson’s Chi-squared tests with Yates’ continuity correction. When the cell counts were small (<5), the *p*-values were calculated using Monte Carlo simulations with 2000 replications. Statistical significance was defined as *p* < 0.05. All analyses were performed using the statistical software R version 4.5.1.

## 3. Results

Thirty women underwent obstetrical hysterectomy among 4200 deliveries over the five-year study period, yielding an incidence of 7.14 per 1000 deliveries (0.71%). Seventeen procedures were electively scheduled to manage PAS, while 13 were performed urgently to control severe hemorrhage. Indications for urgent surgery included uncontrolled bleeding from PAS and refractory uterine atony. Among the urgent procedures, 12 were standard obstetrical hysterectomies and 1 was a Type C radical abdominal hysterectomy according to the Querleu–Morrow classification (as described in Section 2.3), performed for FIGO grade 3c placenta percreta with parametrial and uterine arterial invasion [13,14].

### 3.1. Maternal Demographics and Obstetric History

Maternal age did not differ significantly between groups (36.1 ± 6.1 vs. 37.9 ± 6.8 years; *p* = 0.442). Women in the elective group had higher parity (median 3 [IQR 3–3] vs. 1 [1,2]; *p* = 0.002) and more previous cesarean deliveries (median 2 [IQR 2–2] vs. 0 [0–1]; *p* = 0.002). The prevalence of assisted reproduction (*p* = 0.110), uterine myomas (*p* = 0.564), gestational diabetes (*p* = 0.234), hypertension (*p* = 0.674), preeclampsia (*p* = 0.691), cholestasis (*p* > 0.999), fetal growth restriction (*p* = 0.686), and smoking (*p* > 0.999) did not differ significantly. Rates of twin gestation were also comparable (*p* = 0.582). These results are shown in Table 1.

### 3.2. Obstetric and Placental Findings

Placenta previa was more frequent in the elective cohort (*p* = 0.027), and the distribution of PAS grades differed significantly between groups (*p* = 0.047). Fourteen of seventeen elective hysterectomies and eight of thirteen urgent hysterectomies were performed at ≥34 weeks’ gestation; this difference was not statistically significant (*p* = 0.231). Mean placental weight was comparable (537 ± 118 vs. 554 ± 205 g; *p* = 0.788). Uterine atony occurred exclusively in urgent cases (*p* < 0.001). Placental abruption and uterine rupture were uncommon and barely did not reach the prespecified level statistical significance (*p* = 0.058 and *p* = 0.076, respectively). These results are reported in Table 2.

### 3.3. Perioperative Outcomes

Operating times were similar (159 ± 33 vs. 163 ± 54 min; *p* = 0.628). Length of hospitalization did not differ (median 5 [IQR 5–7] vs. 5 [5,6] days; *p* = 0.861). Preoperative hemoglobin was comparable (12.02 ± 1.25 vs. 11.71 ± 1.01 g/dL; *p* = 0.459), as was postoperative hemoglobin (9.09 ± 0.80 vs. 8.83 ± 1.34 g/dL; *p* = 0.536). Salpingo-oophorectomy was performed in two patients in each group. Uterine fundal massage and/or carbetocin administration did not differ significantly (*p* = 0.494). No internal iliac artery ligation was performed. Bakri balloon tamponade was used in eight urgent cases and in no elective cases (*p* < 0.001). One urgent patient with FIGO grade 3c placenta percreta required Type C radical abdominal hysterectomy because of persistent hemorrhage from extensive parametrial and uterine vascular invasion [19]. In that previously reported case, tranexamic acid, fibrinogen concentrate, and prothrombin complex concentrate were administered, and topical hemostatic adjuncts (oxidized regenerated cellulose powder and a hemostatic patch) were used for diffuse operative bleeding [19]. Because tranexamic acid and topical hemostatic agents were not systematically captured in the study dataset, no valid between-group comparison of these treatments can be provided. Median red-cell transfusion requirements were similar (3 [IQR 2–3] vs. 3 [2–4] units; *p* = 0.877), as was fibrinogen concentrate use (*p* = 0.927). These results are reported in Table 3.

### 3.4. Maternal Complications

No maternal deaths occurred. Bladder injury was numerically more frequent in patients who underwent elective procedures (5/17 vs. 1/13; *p* = 0.219), as was ureteral injury (3/17 vs. 0/13; *p* = 0.255), although neither difference was statistically significant. Disseminated intravascular coagulation developed in two urgent cases (*p* = 0.178). Other adverse events, including transfusion-related acute lung injury, wound infection, postoperative fever, paralytic ileus, reoperation, and ICU admission, showed no significant group differences. The overall number of complications was similar (median 1 [IQR 1–2] in both groups; *p* = 0.742). These results are reported in Table 4.

### 3.5. Neonatal Outcomes

Mean birth weight was similar (2424 ± 575 vs. 2335 ± 919 g; *p* = 0.763), as was female infant proportion (*p* = 0.921). Median Apgar scores at 1 and 5 min were comparable (7 [IQR 5–8] vs. 7 [6–8]; *p* = 0.543 for 1 min; and 8 [7–9] vs. 9 [8,9]; *p* = 0.384 for 5 min). NICU admission rates were not significantly different (*p* = 0.676). These results appear in Table 5.

## 4. Discussion

This retrospective analysis of 30 obstetric hysterectomies included both electively scheduled procedures for antenatally diagnosed placenta accreta spectrum (PAS) (n = 17) and urgent hysterectomies performed for severe hemorrhage (12 standard and 1 radical hysterectomy) at a Greek tertiary referral hospital. The cohorts differed in baseline obstetric risk. Namely, the elective group had higher parity and more previous cesarean deliveries, and placenta previa was more frequent, consistent with the established association between previa, prior cesareans, and PAS. Our finding echoes that of Silver et al., who observed a sharp rise in PAS with increasing numbers of prior cesareans, especially when placenta previa is present (e.g., among women with previa, the risk of accreta rises from 3% with the first cesarean to 67% with ≥5), and aligns with ACOG guidance emphasizing prenatal recognition and delivery at level III/IV centers [16,22].

As expected for confirmed PAS, cesarean delivery was more common in the elective cohort, whereas spontaneous labor occurred only in urgent cases. This pattern parallels multicenter PAS data from Morlando et al., who reported that one-third of women with suspected PAS still deliver emergently and that intensive-therapy/ICU admission is more frequent after emergency delivery [23]. Although our series did not show a statistically significant difference in ICU admissions, likely owing to sample size, the direction of effect is compatible with Morlando and colleagues’ observations (about 12% difference).

Uterine atony was confined to urgent cases, and Bakri balloon tamponade was used only in the urgent group. These cases illustrate escalation from medical and nonsurgical measures to definitive surgery when hemorrhage remained uncontrolled. One study reported that uterine atony was the leading indication for emergency peripartum hysterectomy and that the average EBL exceeded 3 L; broader reviews have highlighted atony as a dominant driver of “crash” hysterectomy [24]. Current FIGO recommendations support early intravenous tranexamic acid for clinically diagnosed postpartum hemorrhage as soon as possible and within 3 h of birth, using 1 g intravenously over approximately 10 min and a second 1 g dose if bleeding continues after 30 min or restarts within 24 h, a protocol well-established in our clinical practice [21,25]. Conwell et al. further emphasize tranexamic acid, targeted fibrinogen replacement when indicated, and selected topical hemostatic adjuncts as components of contemporary obstetric hemorrhage management, which aligns with the protocols of anesthesiology team in our hospital [21]. In our study, however, tranexamic acid and topical hemostatic-agent exposure were administered in all cases.

By contrast, operating time, length of stay, perioperative hemoglobin change, and transfusion units were similar between our elective and urgent groups. This contrasts somewhat with single- and multicenter PAS reports in which planned delivery reduces blood loss and resource utilization. Shamshirsaz et al. found worse maternal and neonatal outcomes after urgent versus planned PAS deliveries in a dedicated multidisciplinary program [8].; Morlando et al. likewise noted higher ICU utilization with emergency delivery, and meta-analytic data show fewer transfused units and shorter hospitalization with planned care [23]. The absence of a difference in our sample likely reflects limited power and the fact that many urgent cases were managed rapidly and effectively under a standardized protocol based on the international guidelines [26,27].

Furthermore, our finding that PAS grade distribution differed between groups is clinically coherent. A relevant study reports that increta/percreta carry markedly higher odds of surgical injury and critical care use than accreta or no PAS, reinforcing the central role of invasion depth alongside planning status [28]. Moreover, when PAS was identified in advance and managed electively in our series, outcomes remained consistently favorable despite higher baseline risk markers. Erfani et al. similarly found that patients with unexpected PAS incurred greater blood loss (median 2.4 L versus 1.7 L; *p* = 0.04) and required more transfusion units (4 versus 2; *p* = 0.03) even though their anticipated group exhibited more extensive invasion, a benefit they attributed to multidisciplinary preparation [9]. A patient with emergent placenta previa with placenta percreta (FIGO grade 3E) at 37 + 6 weeks’ gestation exhibited invasion into the anterior and lateral parametria and the urinary bladder. A radical hysterectomy was required for hemorrhage control, during which disseminated intravascular coagulation (DIC) developed. The postoperative course was protracted over six months and necessitated four reoperations for recurrent hemorrhage and complications related to bladder injury, including intraperitoneal urine leakage (uroperitoneum) [13].

We observed no maternal deaths and found similar overall complication rates in both groups. Bladder and ureteral injuries were more common in elective cases, reflecting the concentration of complex percreta managed electively, although this difference did not reach statistical significance. Contemporary obstetric hysterectomy series highlight the magnitude of urologic risk. Orsi et al. reported a 17.7% rate of urologic injuries, predominantly to the bladder, in pregnancy-related hysterectomies, with both the number of prior cesarean deliveries and the estimated blood loss independently associated with injury risk factors present across our cohorts [29]. Uncontrolled bleeding from the infundibulopelvic ligament led to unilateral adnexectomy in four cases (14% of which 2 were elective and 2 were urgent OHs). A synthesis of six studies on peripartum hysterectomy documented comparable intraoperative complication rates including cystostomy 6–29%, ureteral injury 2–7%, oophorectomy 6%, and reoperation ranged between 4 and 33% of cases. In two cases of elective hysterectomy to treat placenta percreta, patients developed long-term vesicovaginal fistulae. Both were managed successfully with continuous Foley catheter drainage for two weeks. These findings underscore the need for immediate urologic support or the presence of a specialized gynecologic oncology surgeon at all obstetric hysterectomies, whether planned or unplanned [30].

Neonatal outcomes were similar in both groups. Birth weight, Apgar scores, and NICU admission rates showed no significant differences, likely because most deliveries, including those affected by PAS, occurred at or after 34 weeks of gestation, and many elective deliveries took place at approximately 36 weeks. Shamshirsaz et al. found that urgent delivery in PAS cohorts was linked to poorer neonatal indicators such as lower five-minute Apgar scores and more frequent respiratory distress, which supports planning delivery in the late preterm period when possible [8]. In a systematic review, Lucidi et al. found that emergency delivery in pregnancies at high risk for PAS increased the odds of transfusion of more than four units, disseminated intravascular coagulation, intensive care unit admission, and adverse neonatal outcomes including NICU admission and five-minute Apgar scores below seven [31]. In a separate systematic review and meta-analysis, Lucidi et al. reported that women undergoing surgery for PAS face a particularly high incidence of urologic complications, primarily bladder injury (cystotomy), especially those with placenta percreta or those requiring emergency hysterectomy [32].

The lack of significant differences in neonatal separation in our series probably reflects later gestational ages at delivery and the small sample size. Nevertheless, the tendency toward greater need for prematurity-related support in urgent cases is consistent with these reports and with the large single-center Vietnamese experience reported by Thang et al., who observed lower gestational ages and higher transfusion requirements following emergent delivery [33].

Resource readiness remains critical in emergency hysterectomy. A Cameroonian five-year review found that absence of preoperative transfusion and operations > 2 h independently worsened outcomes, emphasizing the need for robust blood bank pathways and surgical efficiency, elements that were present in our service (no internal iliac ligations required; Bakri tamponade targeted to atony) [2]. Moreover, internal iliac artery ligation (IIAL) was not performed in any of our 30 hysterectomy cases as it was unnecessary; resuscitative endo-vascular balloon occlusion of the aorta was not performed either because our hospital lacks an interventional radiology department [34]. The absence of internal iliac ligation in this series should therefore not be interpreted as evidence against the technique; when indicated, IIAL remains safe and effective. A recent 2026 retrospective study of 61 gynecologic and obstetric cases, including 18 obstetric indications, reported 96.7% primary hemostatic success after internal iliac artery ligation and no clinically detected ischemic complications during six months of follow-up, reinforcing its continued use by experienced pelvic surgeons [35]. In our radical hysterectomy case, profound hemodynamic instability precluded high-risk dissection of the great vessels, prompting direct ligation of the bleeding parametrial pedicles instead.

Contemporary surgical management of placenta accreta spectrum (PAS) spans from conservative, uterus-sparing resections to radical peripartum hysterectomy for the most invasive lesions. In a multicenter Argentine cohort of 452 women with PAS, Palacios-Jaraquemada et al. demonstrated that a one-step resective–reconstructive technique, consisting of systematic en bloc excision of the invaded myometrial segment and adherent placenta, layered repair of the uterine wall, and targeted ligation of vesical–uterine and colpo-uterine vessels, with bilateral internal iliac artery ligation in selected cases, achieved uterine preservation in 81 percent of patients and a median intraoperative blood loss of 500 mL (IQR) [36]. A meta-analysis of 16 studies which included 2300 women with PAS demonstrated that conservative management of placenta accreta spectrum is associated with significantly lower estimated blood loss and reduced genitourinary injury rates compared to cesarean hysterectomy [37]. In parallel, intraoperative cell salvage has become integral to high-risk obstetric surgery, as it reduces allogeneic transfusion rates without added morbidity [38]. Reflecting these advances, the FIGO 2018 consensus recommends a standardized, multidisciplinary protocol for PAS management, incorporating precise prenatal diagnosis (ultrasound and MRI), elective delivery planning, intraoperative vascular control (including internal iliac artery ligation and autologous blood recovery), and coordinated postoperative care [3,27]. These data support individualized PAS management at experienced centers rather than a universal hysterectomy-first approach.

The recently developed modified radical peripartum cesarean hysterectomy, also known as the Soleymani–Alazzam–Collins technique, provides a reproducible, stepwise approach to treating FIGO grade 3b–3c disease, for which uterine preservation is contraindicated. After bilateral internal iliac artery ligation to devascularize the pelvis, a high transverse uterine incision above the invasion site allows for fetal delivery; the uterus and adherent placenta are then removed en bloc along avascular planes, and the vaginal cuff is closed in layers [39]. In a retrospective UK series of 24 women, this method halved median blood loss and significantly reduced intensive care admissions compared with conventional hysterectomy without prolonging operative time [39].

The strengths of this study include histopathologic confirmation, the inclusion of both placenta accreta spectrum (PAS) and atony indications, and standardized multidisciplinary management. Its primary limitations include the single-center design and a small sample size. This likely underpowered the detection of differences in infrequent outcomes (e.g., ICU admission, specific organ injury) and may explain why transfusion requirements and length of stay (LOS) did not differ significantly, contrasting with literature trends that favor elective PAS care. Furthermore, selection differences between planned PAS procedures and those performed for unplanned hemorrhage limit broader inference. Moreover, multivariate regressions that would adjust for baseline confounders and confounding by indication were not possible due to the sample size. Therefore, our analyses remain exploratory.

Institutionally, our center lacks a dedicated team for uterine-sparing techniques and does not have access to intraoperative autologous cell salvage equipment. Detailed patient-level information regarding activation of the massive transfusion protocol, FFP and other individual blood-component requirements, and additional hemostatic therapies administered by the anesthesiology team was not consistently retrievable from the patients’ records. Despite the limited sample size, obstetric hysterectomy is a rare intervention reserved for severe conditions; therefore, reporting 30 consecutive cases over a five-year period provides a clinically meaningful single-center experience, even if insufficiently powered for robust subgroup analysis.

## 5. Conclusions

In this single-center experience, planned and urgent obstetric hysterectomies represented distinct clinical pathways. Planned procedures, predominantly performed for antenatally diagnosed PAS, enabled multidisciplinary preparation and controlled surgical management, whereas urgent procedures were undertaken for acute obstetric hemorrhage of heterogeneous etiology. Although the limited cohort size limits inference, our findings underscore the importance of antenatal recognition of high-risk placentation, multidisciplinary preparation, availability of experienced surgical teams, and institutional readiness for major obstetric hemorrhage. Neonatal outcomes were broadly similar, likely because most deliveries occurred ≥34 weeks, but trends in the literature and our cohort suggest that emergency deliveries carry higher neonatal risk largely through prematurity. Preventing avoidable emergencies through early diagnosis, meticulous multidisciplinary planning, and controlled delivery timing remains central to optimizing both maternal and neonatal safety.

## Figures and Tables

**Table 1:** Maternal demographics and obstetric history by elective and urgent hysterectomy (N = 30).

Variable	Elective (n = 17)	Urgent (n = 13)	*p*-Value
Age in years; mean (SD)	36.06 (6.11)	37.92 (6.75)	0.442 §
Uterine myomas; n (%)	1 (5.9%)	2 (15.4%)	0.564 †
In vitro fertilization; n (%)	3 (17.6%)	6 (46.2%)	0.110 †
Parity; Count (IQR)	3 (3–3)	1 (1–2)	0.002 ‡
Previous cesarean sections; count (IQR)	2 (2–2)	0 (0–1)	0.002 ‡
Normal labor; n (%)	0 (0.0%)	5 (38.5%)	0.009 †
Cesarean section; n (%)	17 (100.0%)	8 (61.5%)	0.013 †
Twin gestation; n (%)	1 (5.9%)	2 (15.4%)	0.582 †
Fetal growth restriction; n (%)	6 (35.3%)	3 (23.1%)	0.686 †
Gestational diabetes; n (%)	3 (17.6%)	1 (7.7%)	0.234 †
Hypertension; n (%)	4 (23.5%)	2 (15.4%)	0.674 †
Preeclampsia; n (%)	6 (35.3%)	3 (23.1%)	0.691 †
Cholestasis; n (%)	2 (11.8%)	1 (7.7%)	>0.999 †
Smoking; n (%)	2 (11.8%)	2 (15.4%)	>0.999 †

Notes: SD = standard deviation. IQR = interquartile range. § Welch two-sample *t*-test. † Pearson’s Chi-squared test with simulated *p*-value. ‡ Wilcoxon rank-sum test with continuity correction. Percentages are calculated within each study group.

**Table 2:** Obstetric and placental findings for elective and urgent hysterectomy (N = 30).

Variable	Elective (n = 17)	Urgent (n = 13)	*p*-Value
Placenta previa; n (%)	12 (70.6%)	3 (23.1%)	0.027 †
Placenta accreta spectrum; n (%)			0.047 ‡
Accreta	0 (0.0%)	2 (15.4%)	
Increta	5 (29.4%)	1 (7.7%)	
Percreta	12 (70.6%)	2 (15.4%)	
Abruptio placenta; n (%)	1 (5.9%)	0 (0.0%)	0.058 ‡
Uterine rupture; n (%)	0 (0.0%)	3 (23.1%)	0.076 ‡
Uterine atony; n (%)	0 (0.0%)	9 (69.2%)	<0.001 ‡
Gestational age (≥34 weeks); n (%)	14 (82.4%)	8 (61.5%)	0.231 ‡
Placental weight (g); mean (SD)	537 (118)	554 (205)	0.788 ‡

Notes: SD = standard deviation. ‡ Pearson’s Chi-squared test with simulated *p*-value. † Pearson’s Chi-squared test with Yates’ continuity correction. Percentages are calculated within each study group.

**Table 3:** Perioperative outcomes following elective and urgent hysterectomy (N = 30).

Variable	Elective (n = 17)	Urgent (n = 13)	*p*-Value
Operating time in minutes; mean (SD)	159 (33)	163 (54)	0.628 §
Days of hospitalization; Count (IQR)	5 (5–7)	5 (5–6)	0.861 ‡
Preoperative hemoglobin; mean (SD)	12.02 (1.25)	11.71 (1.01)	0.459 §
Postoperative hemoglobulin; mean (SD)	9.09 (0.80)	8.83 (1.34)	0.536 §
Total hysterectomy; n (%)	16 (94.1%)	13 (100.0%)	>0.999 †
Salpingo-oophorectomy; n (%)	2 (11.8%)	2 (15.4%)	>0.999 †
Uterine fundal massage and/or carbetocin; n (%)	15 (88.2%)	13 (100.0%)	0.494 †
Bakri balloon tamponade; n (%)	0 (0.0%)	8 (61.5%)	<0.001 †
Type C radical hysterectomy (Querleu–Morrow); n (%)	0 (0.0%)	1 (7.7%)	0.433 †
Red-cell transfusion (units); median (IQR)	3 (2–3)	3 (2–4)	0.877 ‡
Human fibrinogen concentrate; n (%)	6 (35.3%)	6 (46.2%)	0.927 †

Notes: SD = standard deviation. IQR = interquartile range. § Welch two-sample *t*-test. † Pearson’s Chi-squared test with simulated *p*-value. ‡ Wilcoxon rank-sum test with continuity correction. Percentages are calculated within each study group.

**Table 4:** Maternal complications following elective and urgent hysterectomy (N = 30).

Variable	Elective (n = 17)	Urgent (n = 13)	*p*-Value
Bladder injury; n (%)	5 (29.4%)	1 (7.7%)	0.219 †
Ureter injury; n (%)	3 (17.6%)	0 (0.0%)	0.255 †
Disseminated intravascular coagulation; n (%)	0 (0.0%)	2 (15.4%)	0.178 †
TRALI; n (%)	0 (0.0%)	1 (7.7%)	0.433 †
Wound infection; n (%)	0 (0.0%)	1 (7.7%)	0.423 †
Post-op fever; n (%)	2 (11.8%)	5 (38.5%)	0.198 †
Paralytic Ileus; n (%)	1 (5.9%)	1 (7.7%)	>0.999 †
Reoperation; n (%)	1 (5.9%)	1 (7.7%)	>0.999 †
Intensive care unit admission; n (%)	12 (70.6%)	11 (84.6%)	0.424 †
Complications; count (IQR)	1 (1–2)	1 (1–2)	0.742 ‡

Notes: SD = standard deviation. IQR = interquartile range. TRALI = transfusion-related acute lung injury. † Pearson’s Chi-squared test with simulated *p*-value. ‡ Wilcoxon rank-sum test with continuity correction. Percentages are calculated within each study group.

**Table 5:** Neonatal outcomes by elective and urgent hysterectomy (N = 30).

Variable	Elective (n = 17)	Urgent (n = 13)	*p*-Value
Fetal birth weight in grams; mean (SD)	2424 (575)	2335 (919)	0.763 §
Female infant; n (%)	9 (52.9%)	8 (61.5%)	0.921 †
Apgar score at 1 min; median (IQR)	7 (5–8)	7 (6–8)	0.543 ‡
Apgar score at 5 min; median (IQR)	8 (7–9)	9 (8–9)	0.384 ‡
NICU admission; n (%)	9 (52.9%)	5 (38.5%)	0.676 †

Notes: SD = standard deviation. IQR = interquartile range. NICU = neonatal intensive care unit. § Welch two-sample *t*-test. † Pearson’s Chi-squared test with Yates’ continuity correction. ‡ Wilcoxon rank-sum test with continuity correction. Percentages are calculated within each study group.

## Data Availability

Data available upon reasonable request.
